# Binary-state scanning probe microscopy for parallel imaging

**DOI:** 10.1038/s41467-022-29181-z

**Published:** 2022-03-17

**Authors:** Gwangmook Kim, Eoh Jin Kim, Hyung Wan Do, Min-Kyun Cho, Sungsoon Kim, Shinill Kang, Dohun Kim, Jinwoo Cheon, Wooyoung Shim

**Affiliations:** 1 Department of Materials Science and Engineering, Yonsei University, Seoul, 03722 Republic of Korea; 2 Center for Multi-Dimensional Materials, Yonsei University, Seoul, 03722 Republic of Korea; 3 Center for NanoMedicine, Institute for Basic Science (IBS), Seoul, 03722 Republic of Korea; 4 KIURI Institute, Yonsei University, Seoul, 03722 Republic of Korea; 5 Department of Physics and Astronomy, and Institute of Applied Physics, Seoul National University, Seoul, 08826 Republic of Korea; 6 School of Mechanical Engineering, Yonsei University, Seoul, 03722 South Korea; 7 National Center for Optically-assisted Mechanical Systems, Yonsei University, Seoul, 03722 South Korea; 8 Graduate Program of Nano Biomedical Engineering (NanoBME), Advanced Science Institute, Yonsei University, Seoul, 03722 Republic of Korea; 9 Department of Chemistry, Yonsei University, Seoul, 03722 Republic of Korea

**Keywords:** Scanning probe microscopy, Imaging techniques

## Abstract

Scanning probe microscopy techniques, such as atomic force microscopy and scanning tunnelling microscopy, are harnessed to image nanoscale structures with an exquisite resolution, which has been of significant value in a variety of areas of nanotechnology. These scanning probe techniques, however, are not generally suitable for high-throughput imaging, which has, from the outset, been a primary challenge. Traditional approaches to increasing the scalability have involved developing multiple probes for imaging, but complex probe design and electronics are required to carry out the detection method. Here, we report a probe-based imaging method that utilizes scalable cantilever-free elastomeric probe design and hierarchical measurement architecture, which readily reconstructs high-resolution and high-throughput topography images. In a single scan, we demonstrate imaging with a 100-tip array to obtain 100 images over a 1-mm^2^ area with 10^6^ pixels in less than 10 min. The potential for large-scale tip integration and the advantage of a simple probe array suggest substantial promise for our approach to high-throughput imaging far beyond what is currently possible.

## Abstract (web summary)

High-throughput imaging has generally been challenging for scanning probe microscopy techniques. Here, the authors introduce binary-state scanning probe microscopy, which uses a cantilever-free elastomeric probes and a hierarchical measurement architecture for parallel topography imaging.

## Introduction

From the invention of the topografiner^1^, the development of scanning probe microscopy (SPM) techniques, i.e., scanning tunnelling microscopy^2^ and atomic force microscopy (AFM)^3^, has accelerated the discovery of significant findings in the field of nanoscience and nanotechnology. The unprecedented resolution of this methodology is attributed to the highly confined apex of the physical probe that traces the topography of the surface through the tip-sample interaction^4^. This working principle enables high resolving power over other microscopy techniques but at the same time gives rise to a trade-off in throughput owing to the serial nature of the single probe scanning process.

Fundamentally, the maximum rate of the topographic change that a single probe can record is limited by the bandwidth of the system, and thus, achieving high resolution inevitably entails deterioration in the throughput. Currently, SPM systems usually have a maximum field of view of up to a hundred micrometres, which is insufficient for high-throughput metrology and restricts the potential range of applications. Despite the previous progress in increasing the imaging speed^5–8^, the single probe measurement still compels a narrow field of view, which can be useful in specific cases such as the investigation of biomolecules^9,10^ and hardly fulfils the general demand for high-throughput topography measurement.

To overcome this throughput issue, parallelized SPM harnessing a multiple probe array has been proposed mainly for AFM^11–18^. Parallel AFM usually adopts an integrated probe design in which the individual probe contains an on-chip deflection detector to read the tip-sample interaction^12,15,19^. These approaches, however, were challenged by their complicated fabrication processes, which could involve high-cost and complex manufacturing of the multiple probe array. Optical detection methods such as interferometry^13,14^ and dispersive beam deflection^16^ allow off-chip deflection detection with a simple tip array structure but still require complex external instruments, which makes performing highly parallel measurements practically infeasible. Recently, cantilever-free AFM^18^ impressively demonstrated the massively parallel imaging capability through its scalable probe design, which eliminates the fabrication bottleneck associated with cantilever-based AFM.

From a methodological viewpoint, the imaging architecture of parallel SPM assumes that individual probes in the array function independently. This architecture causes high complexity to construct a parallel detecting system and process high-precision feedback signals of massive probe array, which eventually curtailed the merit of parallel SPM in throughput and resolution. In this regard, the fundamental challenge to be addressed for scalable SPM is efficient measurement architecture fitted into multiple probe operation. Ideally, parallel imaging architecture that enables to reconstruct high-resolution topography through a highly simplified feedback mechanism of a probe array is desirable for high-throughput measurement preserving the imaging capability comparable to conventional single probe SPM.

In this work, we present an efficient parallel imaging mechanism utilizing binary electrical feedback combined with a scalable cantilever-free elastomeric probe array. The topography measurement method, which we term binary-state probe microscopy (BSPM), has the hierarchical architecture divided into a shared *z*-scanner and a parallel probe array. The parallel tip array is attached to the same z-scanner, and thus the tips share the vertical movement, which we refer to as the shared z-distance. In this architecture, the parallel probe array detects contact with the surface only in binary states. These binary contact signals are then reconstructed into high-resolution topography images based on the shared *z*-distance information. This measurement architecture eliminates the need for high-precision detection of the individual probes and thereby dramatically reduces the complexity to fabricate and operate a multiple probe array. Using the BSPM imaging mechanism, we engaged as many as 100 probes and demonstrated high-resolution topography of the surface over an area of one square millimetre. With the potential to achieve both high resolution and high scalability, BSPM can offer a practical alternative for performing multiple topography measurements.

## Results

### Concept of binary-state probe microscopy (BSPM)

**Fig. 1:**
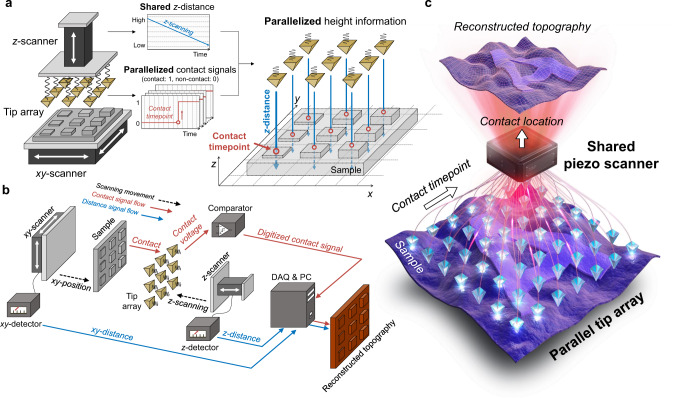
Concept of binary-state probe microscopy (BSPM). **a** Working principle of BSPM measurement. The system reconstructs parallelized height information from parallelized contact signals of the tip array and a shared scanning distance signal of the *z*-scanner using the *xy*-scanner. **b** Schematic of BSPM measurement system. Signal flows of the *xyz*-scanning distances and the parallelized contact signals for topography reconstruction. **c** Conceptual illustration depicting the hierarchical measurement architecture of BSPM with cantilever-free scanning probe array.

The main challenge in designing a feasible multiple probe SPM device is to construct the detection system for the individual probes^20^. To address this challenge, the BSPM method adopts a shared measurement architecture, in which the entire array of tips shares identical scanning distances for reconstructing the topography, and the function of each tip is simplified to detect contact with the surface in a binary fashion. The BSPM system consists of a shared piezo scanner and a parallel tip array (Fig. 1a and Supplementary Fig. 1). The tip array is mounted onto a *z*-scanner and vertically scans while the *xy*-scanner manipulates the position of the sample. During vertical scanning, each tip is brought into contact with the conducting surface at different time points depending on the feature height. The system attains the contact signals of the parallel tip array and the scanning distance of the shared piezo scanner, as shown in Fig. 1b. Then, the contact timepoint of each tip is converted to the specific location of the contact point by referring to the scanning distance of the shared piezo scanner at the same timepoint. In this mechanism, the detection of the tip-sample interaction requires the comparator and a digital input channel to collect the resulting binary contact signal: 1 for contact and 0 for non-contact. This simplified function of the tip enabled by the shared measurement architecture imposes less complexity on the parallelized measurement system than does the conventional SPM method (Fig. 1c).

**Fig. 2:**
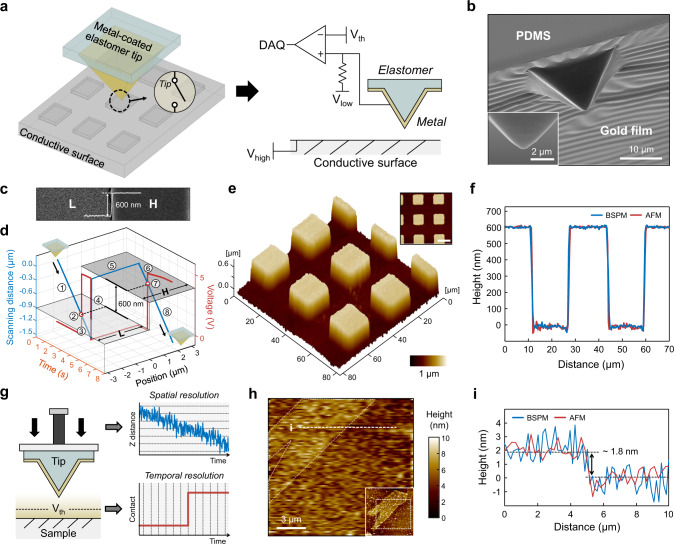
Implementation of BSPM. **a** Schematic illustration depicting the function of the metal-coated elastomer tip. **b** Scanning electron microscopy (SEM) image of the metal-coated elastomer tip. The inset shows the magnified image of the apex of a tip (scale bar: 2 μm). **c** SEM image and line profile of the test sample for the point measurement. **d** Raw data of the point measurement. The red and blue axes show the voltage of the tip and the scanning distance of the piezo actuator, respectively. The circled number indicates the corresponding situation of the tip depicted in Supplementary Fig. 3. Topography image (**e**) and line profile (**f**) of the 16 × 16-μm^2^ square patterns with a thickness of 600 nm measured by BSPM. The inset in **e** shows the 2D mapping of the topography (scale bar: 20 μm). **g** Conceptual illustration showing the spatial resolution of the piezo actuator and the temporal resolution of the tip. **h** Topography of the multilayer graphene sheet measured by BSPM. The inset shows the same region measured by AFM. **i** Line profiles of the multilayer graphene sheet indicated in **h**.

The simplified detection mechanism of the BSPM probe allows a facile fabrication process for the multiple elastomer tip array. The concept of the cantilever-free elastomer tip has been proposed as a scanning probe lithography technique for highly scalable patterning^21–27^. This tip array is readily fabricated by casting polydimethylsiloxane (PDMS) into a pyramid-shaped silicon mould^28^. In BSPM mechanism, each tip functions as a switch that detects the electrical signal (Fig. 2a). By depositing a gold film on each tip (Fig. 2b), we can use these elastomeric tips as imaging probes for the BSPM measurement. Considering that this tip array is an expendable, scalable fabrication of the elastomeric tip array significantly contributes to the feasibility of parallel operation.

To demonstrate the topography measurement capability of BSPM, we first fabricate a metal-coated single elastomeric tip (Fig. 2b and Supplementary Fig. 2), followed by performing a two-point measurement with a height difference of 600 nm (Fig. 2c, d and Supplementary Fig. 3). During two vertical scans (blue line, Fig. 2d), the tip comes in contact with the surface at scanning distances of ‒0.9 μm in the lower region (L) and ‒0.3 μm in the higher region (H), which are distinguishable by a sudden increase in the tip voltage (red line, Fig. 2d). The system correctly synchronizes these two pieces of information (position and voltage signal) and thus attains the height difference between the two measured points (Supplementary Video 1).

Similarly, the 3D topography can be measured by repeating the point measurements (Supplementary Video 2), akin to a tapping mode measurement in conventional AFM. Figure 2e shows the measured topography image of 16 × 16-μm^2^ square patterns with a thickness of 600 nm (Supplementary Fig. 4), which was reconstructed from 65,536 measured points (256 × 256-pixel scan). To evaluate the precision, we measured the line profile of these square patterns. We used the average of five measurements to obtain a high-precision profile. Notably, the imaging quality of the line profile (blue line, Fig. 2f) is comparable to that of commercial AFM (red line, Fig. 2f), verifying the high precision and consistency of the BSPM measurements. The standard deviation of flat regions on the line profile is 9.6 nm for the sample with the roughness of 5.7 nm (Supplementary Fig. 5).

Concerning the vertical resolution, the information provided by the BSPM measurement originates from collecting the contact signal of the tip and detecting the scanning distance of the piezo actuator (Fig. 2g). Ideally, the maximum vertical resolution is limited by the ratio of the spatial resolution (equivalent to the vertical velocity, *v*_piezo,_ of the piezo actuator) to the temporal resolution (equivalent to the sampling rate of the contact signal, *f*_sampling_), as indicated by *v*_piezo_/*f*_sampling_. When we scanned, for example, the point at a speed of 100 μm/s with a sampling rate of 100 kHz, the height resolution was at most 1 nm. In practice, however, we note that the noise level of the detector also limits the precision of the acquired scanning distance. After signal amplification and band-pass filtering to minimize the noise (Supplementary Fig. 6), our prototype BSPM is capable of imaging few-layer graphene (FLG) flakes dispersed on a SiO_2_/Si surface (Supplementary Fig. 7) and of further identifying 1.8-nm FLG (Fig. 2h, i). This vertical resolution is not the fundamental limit of BSPM and could be enhanced by adopting a high-precision piezo actuator and a low-noise data acquisition device. In addition to the data acquisition performance, we noted that the stiffness and resilience of the sample should be considered when estimating the expected vertical resolution. Because the BSPM scans the sample without force feedback, vertical scanning is continued after the electrical detection of a surface is performed. This situation could lead to contact deformation of the sample, although the elastomeric body of the tip minimises such deformation (Supplementary Text 1). For BSPM measurements, it is desirable to choose a sample that is hard enough to prevent contact deformation or recover its shape reversibly after vertical scanning.

### Characterization of the metal-coated elastomer tip

**Fig. 3:**
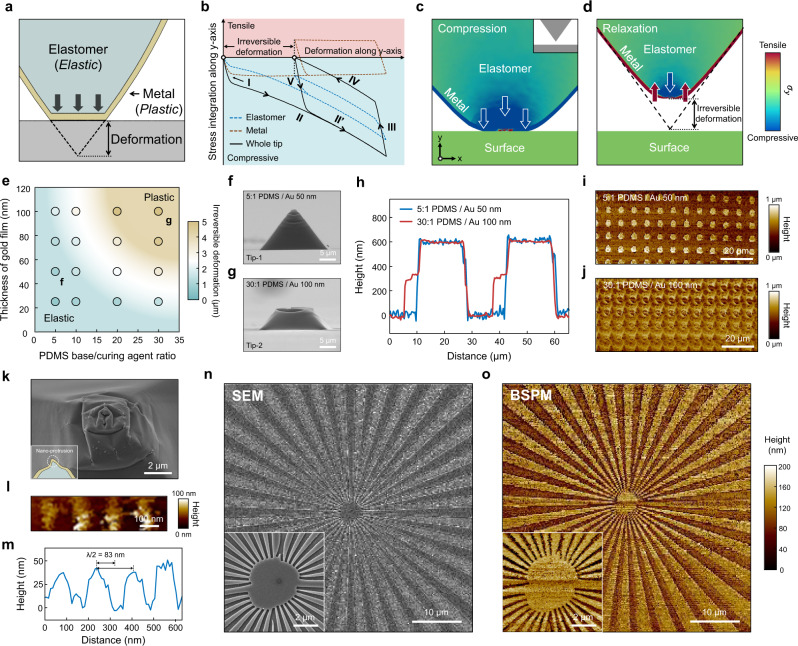
Lateral resolution of BSPM. **a** Schematic illustration depicting the deformation of the metal-coated elastomer tip. **b** Simulated result of the overall stress in the metal-coated elastomeric composite during the compressive-relaxation cycle. Simulated stress distribution in the metal-coated elastomeric composite in the compression (**c**) and relaxation (**d**) steps. The inset in **c** shows the geometry of a metal-coated elastomeric composite at the initial state. **e** Magnitude of the irreversible deformation of the tip depending on the base/curing agent ratio of the PDMS and the thickness of the gold film. SEM images of the metal-coated elastomer tip after the compression-relaxation test under 5:1 PDMS/Au 50 nm (Tip-1, **f**) and 30:1 PDMS/Au 100 nm (Tip-2, **g**). **h**, Line profiles of the 16 × 16-μm^2^ square patterns with a thickness of 600 nm measured by the two tips under different deformation conditions. Topography images of the 4-μm-diameter circle patterns with a thickness of 400 nm measured by tip-1 (**i**) and tip-2 (**j**). **k** SEM image of the wrinkled structure on the tip. Topography (**l**) and line profile (**m**) of the features with a width of 83 nm and a thickness of 40 nm. SEM (**n**) and BSPM (**o**) images of the Siemens star test chart with a thickness of 32 nm. The insets show enlarged views of the images.

BSPM uses cantilever-free tips composed of an elastomeric body coated with a metal film (Fig. 3a). During repeated vertical scanning, the elastomeric body undergoes reversible deformation, while the metal film experiences plastic deformation when the tip is deformed beyond the elastic limit of the metal. This discrepancy between the two components could affect the precision in determining the contact point and, consequently, the lateral resolution.

To analyse this, we performed a finite element method (FEM) simulation to conduct a stress analysis of the metal-coated elastomer composite. Figure 3b presents the overall stress profile during the compression-relaxation cycles (Supplementary Video 3). The stress profile was composed of two distinct cycles: the first irreversible cycle (I–II–III–IV) and the second reversible cycle (V–II′–III–IV) (Fig. 3b). During the compressive steps of the first cycle (I–II), the metal film was deformed plastically while losing resilience towards its initial shape. As a result, when the tip was released from the compressive states (Fig. 3c), the shape recovery of the metal film was limited (III), and the stress state was inverted from compressive to tensile by further relaxation of the elastomeric body. This tensile stress in the metal film compensated for the resilience of the compressed elastomeric body and finally induced irreversible deformation of the tip (IV, Fig. 3d). With this final shape, the stress in the elastomeric body and metal film was balanced. The subsequent compression-relaxation cycle, therefore, resulted in an identical final shape, which made the cycle reversible (V–II′–III–IV). We can conclude that (i) the irreversible deformation results from the competition between the compressive stress (in the elastomeric body) and the tensile stress (in the metal film) and (ii) the metal-coated elastomer tip is capable of reversible vertical scanning, but the lateral resolution may deteriorate due to the widened apex of the tip.

In this sense, to minimize the irreversible deformation, we controlled the stress by modulating the elastomer stiffness and the volume of the metal film. Thus, we varied the base/curing agent ratios and Au film thickness of the tips. We compressed 14-μm-high metal-coated elastomer tips to half their height (7 μm) and measured the height of the recovered tips (Supplementary Fig. 8). The smaller base/curing agent ratio (stiffer) and thinner thickness of the Au film (less volume) led to more reversible deformation of the tip (Fig. 3e). Next, we directly related this reversible deformation with the imaging capability. We prepared two tips: tip-1 with a 5:1 PDMS ratio/50-nm Au (bottom left, Fig. 3e) and tip-2 with a 30:1 PDMS ratio/100-nm Au (top right, Fig. 3e). After one cycle of compression-relaxation, tip-1 (Fig. 3f) exhibited more elastic behaviour than tip-2 (Fig. 3g), which matches well with the FEM. For the line profiles, indeed, tip-2 exhibited artifacts near the feature edge, while tip-1 sharply imaged the feature (Fig. 3h). For 4-μm-diameter circles (Supplementary Fig. 9), the blunt tip (tip-2) hardly distinguished the circles because the size of the features (4 μm) was smaller than the tip diameter (~10 μm) (Fig. 3j); yet, the sharp tip (tip-1) clearly resolved the topography of the features (Fig. 3i). However, we note that excessive stiffness for the PDMS and thickness for the Au film could hamper the mechanical stability (Supplementary Fig. 10)^29,30^. We experimentally found that a tip with a 10:1 base/curing agent ratio and a 50-nm-thick gold film was optimal for measurement.

The deformation of the tip is related to the maximum depth of the feature, as in conventional SPM techniques. Without the deformation, the aspect ratio of a pit that the tip can resolve is $\sqrt{2}$:1, resulting from the shape of the wet-etched silicon mould. Irreversible deformation of the tip causes the mesa on the apex, thereby further limiting the resolvable feature depth. To increase the maximum depth of the feature allowed by the BSPM, sharpening of the silicon mould or an elastomeric tip would be desirable^24,31^. These techniques can potentially improve the lateral resolution of the BSPM tip.

An interesting aspect related to the lateral resolution is the wrinkling of the Au film (Fig. 3k). As discussed, compression of the Au film induces permanent deformation at the apex of the tip, forming wrinkles. Because the wrinkled morphology has nanoscale protrusions, it could act as a sharp tip that increases the lateral resolution, allowing for the resolution of small features down to 83 nm (Fig. 3l, m and Supplementary Fig. 11). This resolution is important because it implies that the BSPM imaging mechanism can achieve finer resolution than optical and stylus profilers, which are also used for large-area surface metrology. Indeed, the resolution of the wrinkled tip is comparable to that of a commercial AFM tip with a sub-50-nm tip diameter. For example, we successfully imaged the Siemens star test chart (50 × 50 μm^2^) with a height of 32 nm (Fig. 3n, o) and, in particular, a dense centre area with a 130 nm lateral resolution (inset of Fig. 3o). We expect that this exceptional resolution made possible by the wrinkled morphology could be beneficial, especially for applications involving nanosheets, such as two-dimensional (2D) materials, as discussed in the following.

### Parallel imaging

**Fig. 4:**
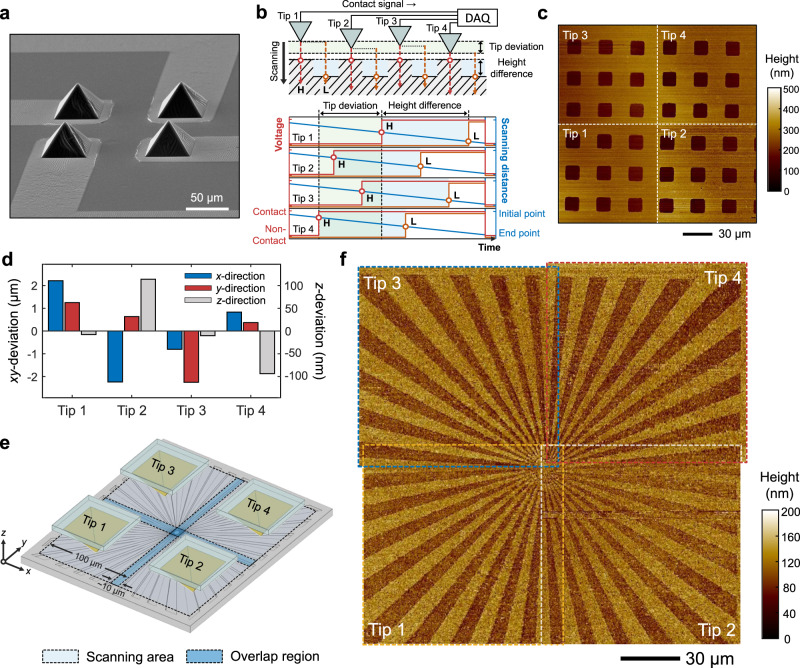
Parallelization of BSPM. **a** SEM image of the 2 × 2 metal-coated elastomer tip array. **b** Schematic illustration and graphs depicting the parallelized measurement using a multiple probe array. **c** Topography images of the square patterns with a thickness of 138 nm obtained through parallelized measurement using the 2 × 2 tip array. **d** Deviation of the 2 × 2 tip array. *xy*- and *z*-deviations indicate the deviation in the position and the height of the tip, respectively. **e** Schematic illustration of parallel imaging with the overlapped scanning area. **f** Parallelized BSPM measurement of the Siemens star test chart using the 2 × 2 tip array.

To probe the capability of BSPM for parallel imaging, we first fabricated a 2 × 2 tip array (Fig. 4a) and performed parallelized measurements. The procedure using multiple tips was similar to that of the single tip measurement. The deformability of the elastomeric tip prevents mechanical crosstalk between the neighbouring tips, making each tip function independently as a single tip (Supplementary Text 2). For parallel imaging, the tip array repeats vertical scanning and lateral translation over the imaging area (Supplementary Video 4). The images result from the combination of (i) the time of contact of the individual tips and (ii) the vertical scanning distance at the time of contact (Fig. 4b and Supplementary Fig. 12). Each tip in the array detects contact to the surface at different time points according to the height of the tip and the feature, followed by the deformation of the elastomeric tip to alleviate the contact damage to the sample. It should be noted that the BSPM measurement obtains topography information when the tip is in contact with the sample. This implies that electrical detection of contact is performed without deformation of the tip. Thus, the difference in the contact deformation of each tip in the array does not affect the measurement, which provides tip-height tolerance in measuring features with high precision. These are the advantages of our contact-based imaging mechanism, which is less sensitive to the tip-height variation in the array.

For proof-of-concept purposes, the pre-defined concave square patterns were imaged by a 2 × 2 tip array (Fig. 4c). The individual image presents consistent topography. Note that in this shared architecture, the height information from the individual tips originated from the scanning distance of the shared *z*-scanner, and therefore, the precision of the parallel imaging was identical to that for single tips. This advantage is significantly beneficial for parallel measurements, eliminating the need to calibrate each tip along the *z*-axis.

As tip position deviation along the *x*- and *y*-axes in the array is inevitable (Fig. 4d), which is inherent to the microfabrication of silicon moulds, one must evaluate whether the tip array can image a large-area surface consisting of continuous lines beyond the field of view of a single tip. To probe this, an imaging experiment was performed with a 2 × 2 tip array, in which each tip was directed to image 100 × 100-μm^2^ subpatterns of the Siemens star test chart, with the results subsequently stitched together to produce an entire image of 190 × 190 μm^2^ (Fig. 4e). This stitching is made by overlapping ~10 × 100 μm^2^ of the edge of each image (dotted lines, Fig. 4f), with the result being a continuous feature of 390-nm-wide lines at the centre (Fig. 4f). Overall, the architecture can accommodate the tip position deviation along the *x*-, *y*-, and z-directions, thereby providing a vehicle for parallel imaging.

**Fig. 5:**
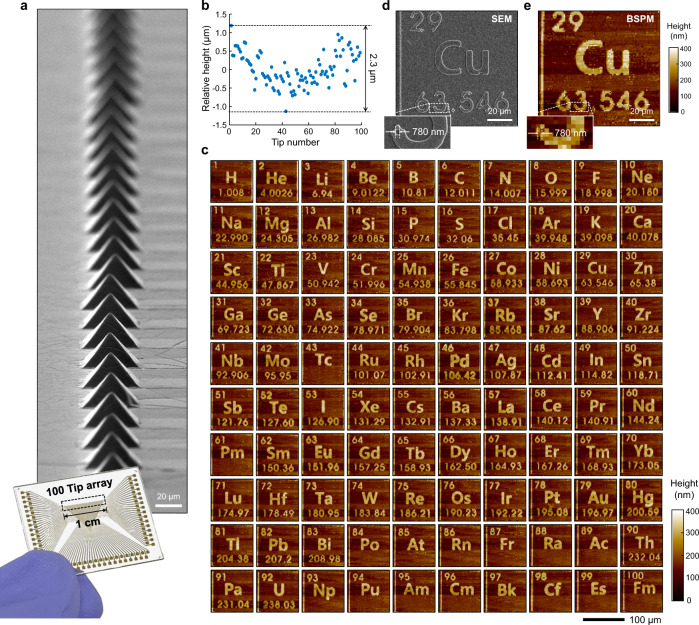
Massively parallelized topography measurement using one hundred tips. **a** SEM image and photograph of the one-hundred-tip array. **b** Deviation of the one-hundred-tip array in the tip height. **c** Topography images of the periodic table patterns measured by the one-hundred-tip array. SEM (**d**) and BSPM (**e**) images of the copper portion of the periodic table patterns.

**Fig. 6:**
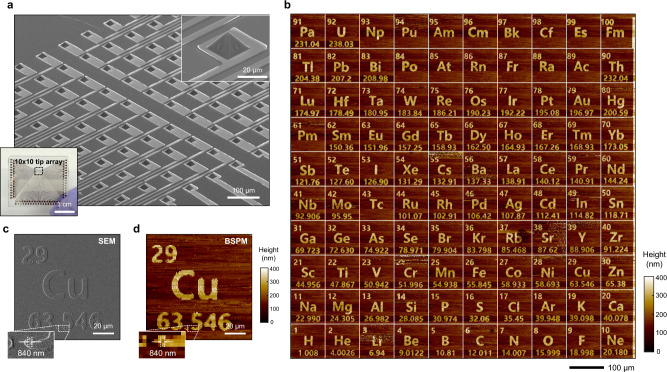
BSPM measurement using the 2D 10 × 10 tip array. **a** SEM images and photograph of the tip array. **b** BSPM measurement of the periodic table patterns. SEM (**c**) and BSPM (**d**) images of the copper portion of the periodic table patterns.

BSPM measurement enables imaging of a surface in a parallel (over 1 mm^2^) and cantilever-free fashion, which dramatically reduces the complexity of the sensing mechanism and array fabrication. In this context, we evaluated the scalability of BSPM by implementing topography measurements using a 100-tip array. We fabricated an array of 100 metal-coated tips with a tip-to-tip distance of 100 μm (Fig. 5a). In this configuration, each individual tip had a maximum field of view of 100 × 100 μm^2^, and the entire tip array covered up to 1 cm × 100 μm (1-mm^2^ area). To perform the measurement, we first aligned the one-hundred-tip array with the surface by tilting the sample stage^32^. We then set a proper vertical scanning range (~2.5 μm) of the tip array to ensure that all tips came in contact with the surface during the measurement. Note that the vertical scanning range of the tip array was slightly larger than that of the single tip because the height of the tip at the centre of the array was shorter than that of the others owing to the curvature of the glass substrate supporting the tip array (2.3 μm, Fig. 5b). The ability to produce the topography image with a tip array was evaluated in the context of imaging pre-patterned elements of the periodic table from 1 Hydrogen to 100 Fermium (Supplementary Fig. 13). Each element of the periodic table occupies a 100 × 100-μm^2^ square, corresponding to the field-of-view of an individual probe. Indeed, 100 representative images generated across the 1-mm^2^ area show the quality and high throughput of this microscope (Fig. 5c). For each image, we conducted a 100 × 100-pixel scan at 589 s for raster scanning and thus provided 10,000 pixels per image, which is similar to that of conventional raster scanning by AFM (typically a 256 × 256-pixel scan). As a result, unlike AFM, BSPM provides 10^6^ pixels for 100 images at a single scan within 10 min with custom software. The vertical precision of parallelized measurement is 16 nm for the sample with the roughness of 3.4 nm (Supplementary Fig. 14). Importantly, the resulting individual element image (Fig. 5e) is an accurate duplication (100 × 100 μm^2^) of the pre-patterned element (Fig. 5d), showing <800-nm lateral resolution, which is nearly equivalent to the single pixel size of 1 μm. Note that the tip diameter, in principle, could be reduced down to sub-50 nm by optimizing the wrinkling conditions (Fig. 3k), but these tip arrays are sufficient to evaluate the potential for large-scale imaging. In addition to the 1D arrangement of tips, parallel tips can be arranged in the 2D 10 × 10 tip array (Fig. 6a). The measurement using the 2D tip array has an identical resolution to that of the 1D tip array (Fig. 6b‒d and Supplementary Fig. 15). The tip fabrication protocol can increase the number of 2D tip arrays by more than 100 tips, which suggests the potential to further improve the throughput of parallel measurement (Supplementary Fig. 16).

Finally, we also explored the potential advantages of this approach that may be useful for the high-throughput investigation of 2D materials. To evaluate this possibility, the 100-tip array scans 100 different areas of exfoliated multilayer graphene sheets on a silicon substrate (individual imaging area of 30 × 30 μm^2^ and total area of 0.09 mm^2^, Supplementary Fig. 17). We found the thinnest multilayer graphene sheet to be 13 nm in thickness (Supplementary Fig. 17b, c). These results confirmed that the shared *z*-detector can indeed provide nanometre-scale resolution even though the tip height varies >2 μm (Fig. 5b). The total time required to image the 100 × 30 × 30 μm^2^ topography was less than 10 min.

## Discussion

BSPM is a scanning-probe-based imaging method that uses cantilever-free tip arrays with the shared measurement architecture, which is multiplexed for large-scale implementation. BSPM can be readily integrated with the conventional piezo actuator and allows resolutions on nanometre-length scales because of the high sampling rate of the contact signal. The feasibility and functionality of BSPM were demonstrated with 100 images over a 1-mm^2^ area with 10^6^ pixels in less than 10 min. This level of scalability is substantially greater than the microscale imaging area of AFM. We note that several challenges remain: (i) establishing electronics to collect and post-process the contact signals from massively parallel tip array, (ii) studying the detailed mechanical contact phenomena of tip arrays that could induce artifacts, and (iii) finding a non-electrical contact sensing mechanism that is capable of imaging an insulating surface. BSPM offers significant advances in terms of scalability and decreased complexity compared to many previous parallel SPM designs and could be implemented with common conventional SPM equipment.

## Methods

### Fabrication of a silicon mould for a metal-coated elastomer tip

A silicon substrate with a 5000 Å oxide layer was sonicated with acetone and isopropyl alcohol. The circular patterns were exposed on the cleaned wafer by photolithography or e-beam lithography. The oxide layer on the silicon substrate was etched with the pre-defined pattern by a 6:1 buffered oxide etchant (J. T. Baker), and then the silicon substrate was etched in 30 wt% potassium hydroxide solution (potassium hydroxide, EP grade, DAEJUNG). After the silicon substrate was etched, the circular patterns resulted in pyramid-shaped holes whose width was equal to the diameter of the circular patterns. The remaining oxide layer was then removed by a 6:1 buffered oxide etchant. The etched silicon wafer was exposed to O_2_ plasma and placed on a vacuum chamber with heptadecafluoro-1,1,2,2-tetrahydrodecyltrichlorosilane (JSI Silicone) diluted in toluene (10 drops for 5-ml toluene in a 20-ml glass vial) to form the anti-adhesion layer.

### Fabrication of a metal-coated elastomer tip

PDMS (Sylgard 184, Dow Corning) with various base/cross-linker ratios was mixed and degassed in a vacuum. PDMS was poured onto the pyramid-shaped silicon mould and cured at 60 °C overnight, with the supporting glass substrate placed on the PDMS. The cured PDMS tip was separated from the silicon mould. To define the signal line on the PDMS tip, it was exposed to O_2_ plasma and then patterned by photolithography using a negative photoresist (DNR-L300, DONGJIN SEMICHEM). Gold film (typically 50-nm Au with a 5-nm Cr adhesion layer) was deposited onto the patterned PDMS tip by thermal evaporation and then lifted off with acetone. In the fabrication of the multiple tip array, unintended gold lines between the adjacent signal lines could be formed through cracks in the photoresist pattern, which occur during thermal evaporation due to thermal expansion of the PDMS layer. This crosstalk line was burned by applying voltage (approximately 5–10 V) between the adjacent signal lines.

## Supplementary information

Supplementary Information

Description of Additional Supplementary Files

Supplementary Video 1

Supplementary Video 2

Supplementary Video 3

Supplementary Video 4

## Data Availability

The data that support the findings of this study are available from the corresponding author upon request.
