# Supplementary material for: Binary-state scanning probe microscopy for parallel imaging
Source: Nat Commun. 2022 Mar 17;13:1438. doi: 10.1038/s41467-022-29181-z (PMC8931021; doi:10.1038/s41467-022-29181-z)
Supplement: Supplementary file 1 — Supplementary Information [file 41467_2022_29181_MOESM1_ESM.pdf]

Supplementary Information

Binary-state scanning probe microscopy for parallel imaging

Gwangmook Kim, Eoh Jin Kim, Hyung Wan Do, Min-Kyun Cho, Sungsoon Kim, Shinill Kang, Dohun Kim, Jinwoo Cheon, Wooyoung Shim*

* E-mail: wshim@yonsei.ac.kr

Table of contents:

Supplementary Methods

Supplementary Figures 1 – 21

Supplementary Table 1

Supplementary Text 1. Contact behaviour of the metal-coated elastomer tip

Supplementary Text 2. Mechanical crosstalk between neighbouring tips

Supplementary Methods

BSPM measurement. The prototyping system consisted of a metal-coated elastomer tip, a tip carrier PCB, comparator circuits (made using TL074CN, Texas Instruments), a DC supplier (E3631A, Agilent), a data acquisition device (PCIe-6363, NI) and a piezo stage (Supplementary Fig. 18). We used the piezo stage of the commercial AFM system (XE-150PPL, Park Systems) in polymer pen lithography mode to control the position of the tip array. The metal-coated elastomer tip array supported by the glass substrate was mounted on the tip carrier PCB. The tip carrier interconnected the tip array to the external comparator circuit and was attached to the z-axis piezo actuator (HH77600718, Park Systems). To connect the tip carrier PCB to the external comparator circuit, we used a hand-wired enamelled copper wire for the single tip and the 2 × 2 tip array and a high-density cable (SH100M-100M Flex Cable, NI) for the one-hundred-tip array. The external comparator circuit are connected to 2 × PCIe-6363 cards by 4 × terminal blocks (SCB-68A, NI) and 4 × cables (SHC68-68-EPM, NI). For single tip and 2 × 2 tip array measurement, we used digital input ports of 1 × PCIe-6363 to receive binary contact signals (Supplementary Fig. 6a). For 100 tip measurement (100 × 1 and 10 × 10 tip array), we used 2 × 32 digital input ports and 2 × 18 analog input ports to receive binary contact signals from 100 tip array using 2 × PCIe-6363. Each DAQ card receives the xyz position of piezostage individually, to synchronize the timing of position information with that of contact signals acquired from each DAQ card (Supplementary Fig. 19).

Detection of the scanning movement of the piezo stage was performed by the built-in displacement detector on the piezo stage. Since the prototype BSPM system used a long-travel-range piezo actuator designed for lithographic purposes (HH77600718, Park systems), the noise level of the displacement detector was approximately 20 nm, which is relatively higher than that of the high-precision piezo actuator. The scanning distance signal in the z-axis can be amplified by an RF preamplifier (SR445A, Stanford Research Systems) to reduce the noise

level of the scanning distance signal (Supplementary Fig. 6b and c). We amplified the scanning distance signal only for the thin sample with a thickness of 10 nm (Fig. 2h and Supplementary Fig. 7) and usually operated the BSPM system without amplification because the amplification decreased the detection range of the scanning distance. The data acquisition device collected the contact signals from the comparator circuits of each tip and the location information of the piezo stage from the signal access module of the AFM stages (Supplementary Fig. 6a).

To implement the BSPM measurement, we first applied a voltage to the sample, which was 40 mV higher than the threshold voltage of the comparator circuit, to detect the contact electrically. We set the measured area, the number of points, the vertical scanning distance and the scanning velocity in the piezo stage control PC and set the sampling rate of the contact signal using the BSPM measurement software (written in MATLAB) in the data acquisition PC. We usually set the scanning velocity to 100 μm/s and sampling rate to 100 kHz. Note that the sampling rate of the digitized contact signal could be increased in our system up to 2 MHz, but 100 kHz is sufficient for 1-nm imaging.

During repetitive vertical scanning, the BSPM measurement software determined the timepoint at which the contact signal changed from the non-contacted (0) to contacted (1) state. When contact occurred, the software recorded the scanning distance of the piezo stage at this timepoint, and the post-processing software (written in MATLAB) calculated the specific location of the contact point by combining the scanning distance of the piezo stage with the initial position of each tip. Post-processing of the measurement usually included levelling the topography and adjusting the data range, similar to the common post-processing steps performed in SPM.

The number of pixels of the image was 256×256 for the single tip and the 2×2 tip array measurement and 100×100 for the one-hundred-tip array measurement. The line profiles were attained by taking the average of the five measurements. The test samples, such as the circle

and square patterns and the Siemens star test chart, were fabricated by etching the silicon oxide layer on the silicon wafer, followed by deposition of 50-nm Au with a 5-nm Cr adhesion layer by thermal evaporation. The few-layer graphene samples on a SiO_2/Si substrate were prepared by mechanical exfoliation from highly oriented pyrolytic graphite (HQ graphene), followed by deposition of 50-nm Au with a 5-nm Cr adhesion layer by thermal evaporation.

Numerical simulation. Finite element analysis (FEA) was used to analyse the deformation of the metal-coated elastomer composite. A static non-linear analysis under the contact condition and plastic deformation was carried out by the open-source FEA software Code_Aster. Note that the purpose of the simulation is to show the typical deformation behaviour of the metal-coated elastomer composite rather than to calculate the actual situation of the BSPM measurement. The geometry and the conditions in the simulation were simplified for facile simulation and intuitive visualization of the simulated results.

In the simulation in Fig. 2b-d, we adopted the 2D plane strain condition. The elastomer was set to have the linear elastic behaviour, and the work hardening effect of the metal film was not considered. The ratio of Young's modulus (E) between the metal and the elastomer $E_{Metal}/E_{Elastomer}$=400, and Poisson's ratio (v) v_{Metal}=0.42 and $v_{Elastomer}$=0.49. The elastic limit of the metal film was set to 0.67%. We simulated the situation where the 14-μm-high metal-coated elastomer composite with the 200-nm-thick metal film was pushed down onto the surface twice with a concept depth of 5 μm. In the simulation of Supplementary Fig. 9a-d, we used the same geometry and condition with variations in the contact distance and Young's modulus of the elastomeric body.

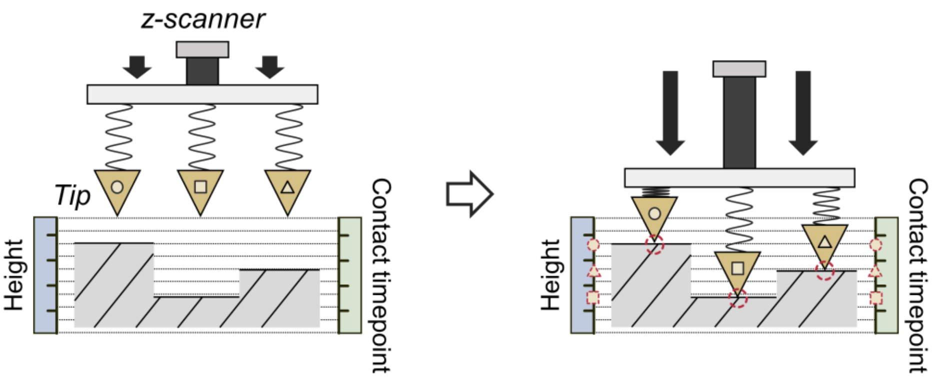

Supplementary Fig. 1 | Illustration depicting the vertical scanning using the parallel tip array.

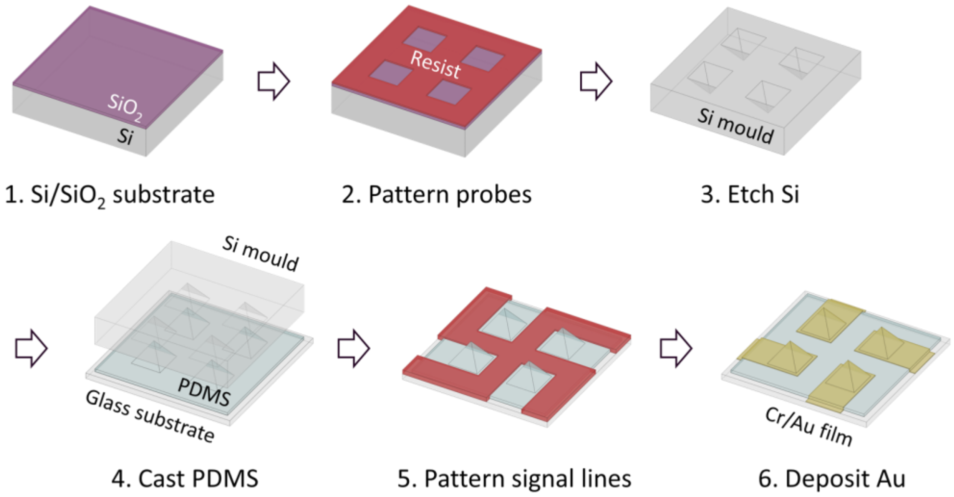

Supplementary Fig. 2 | Fabrication process of the metal-coated elastomer tip.

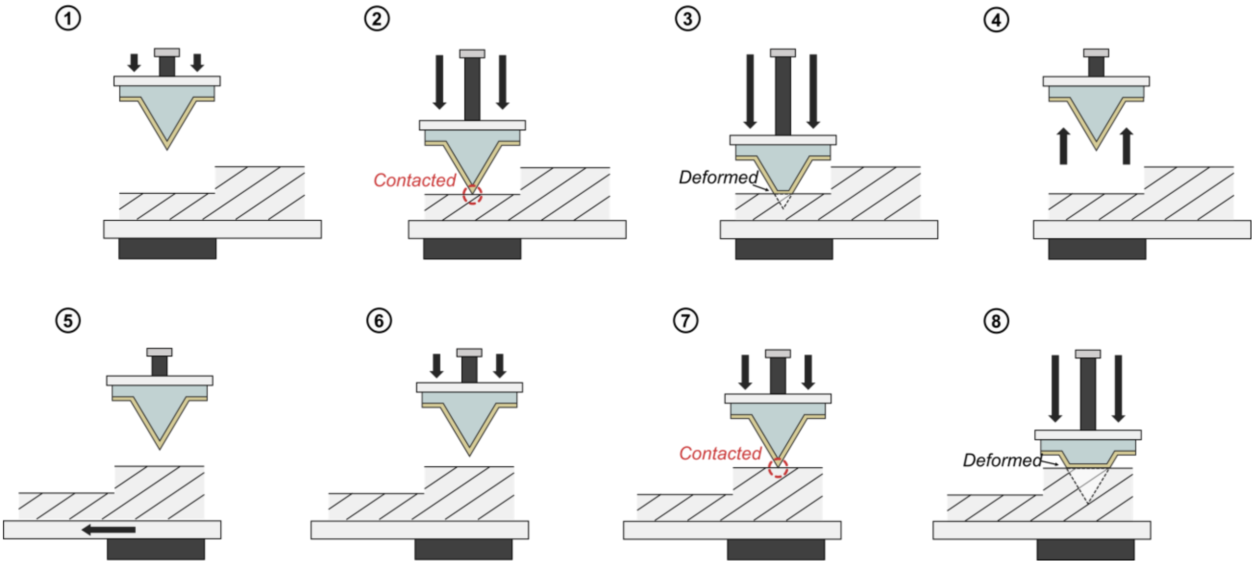

Supplementary Fig. 3 | Illustrations depicting the procedure of point measurement in Fig. 2d.

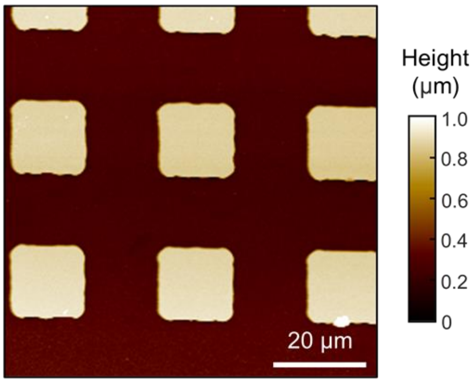

Supplementary Fig. 4 | AFM image of the 16 × 16-μm^2 square patterns with a thickness of 600 nm.

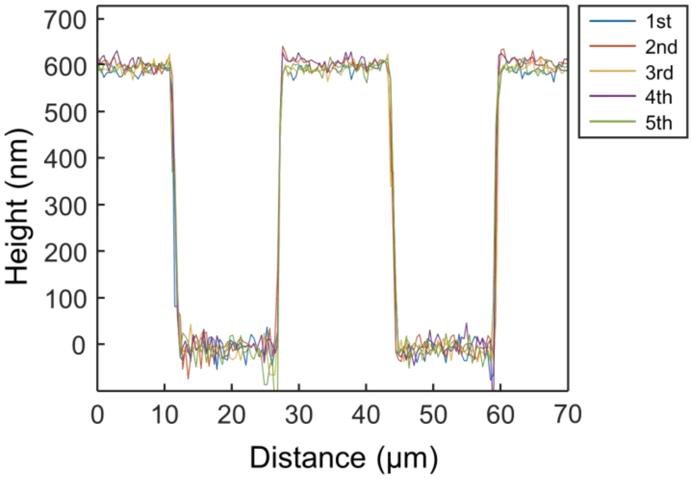

Supplementary Fig. 5 | Raw data of the line profile in Fig. 2f to evaluate measurement precision.

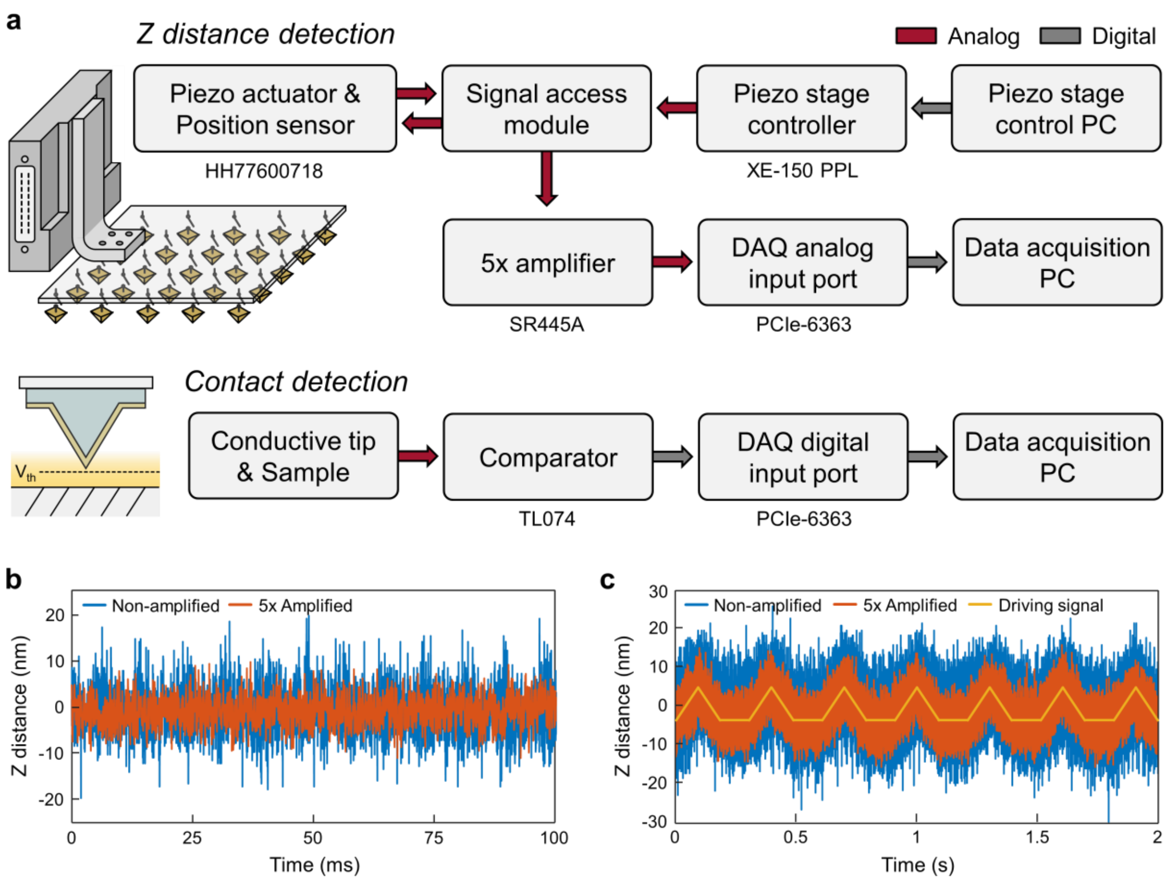

Supplementary Fig. 6 | Signal accessing modules. a, Diagram of the data acquisition instruments for the BSPM measurement. **b-c,** Raw data of the scanning distance signal from the displacement detector on the piezo actuator under the stationary (**b**) and moving (**c**) conditions.

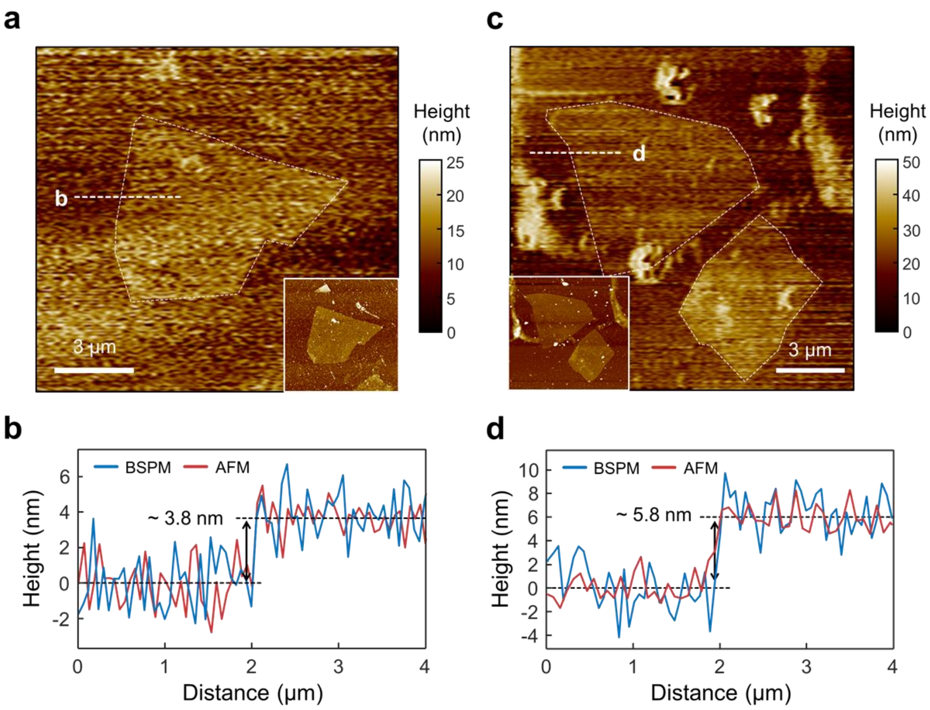

Supplementary Fig. 7 | BSPM measurement of the multilayer graphene sheets. a-b, Topography (**a**) and line profile (**b**) of the 3.8-nm-thick multilayer graphene sheet. The inset in **a** shows the same region measured by AFM. **c-d,** Topography (**c**) and line profile (**d**) of the 5.8-nm-thick multilayer graphene sheet. The inset in **c** shows the same region measured by AFM.

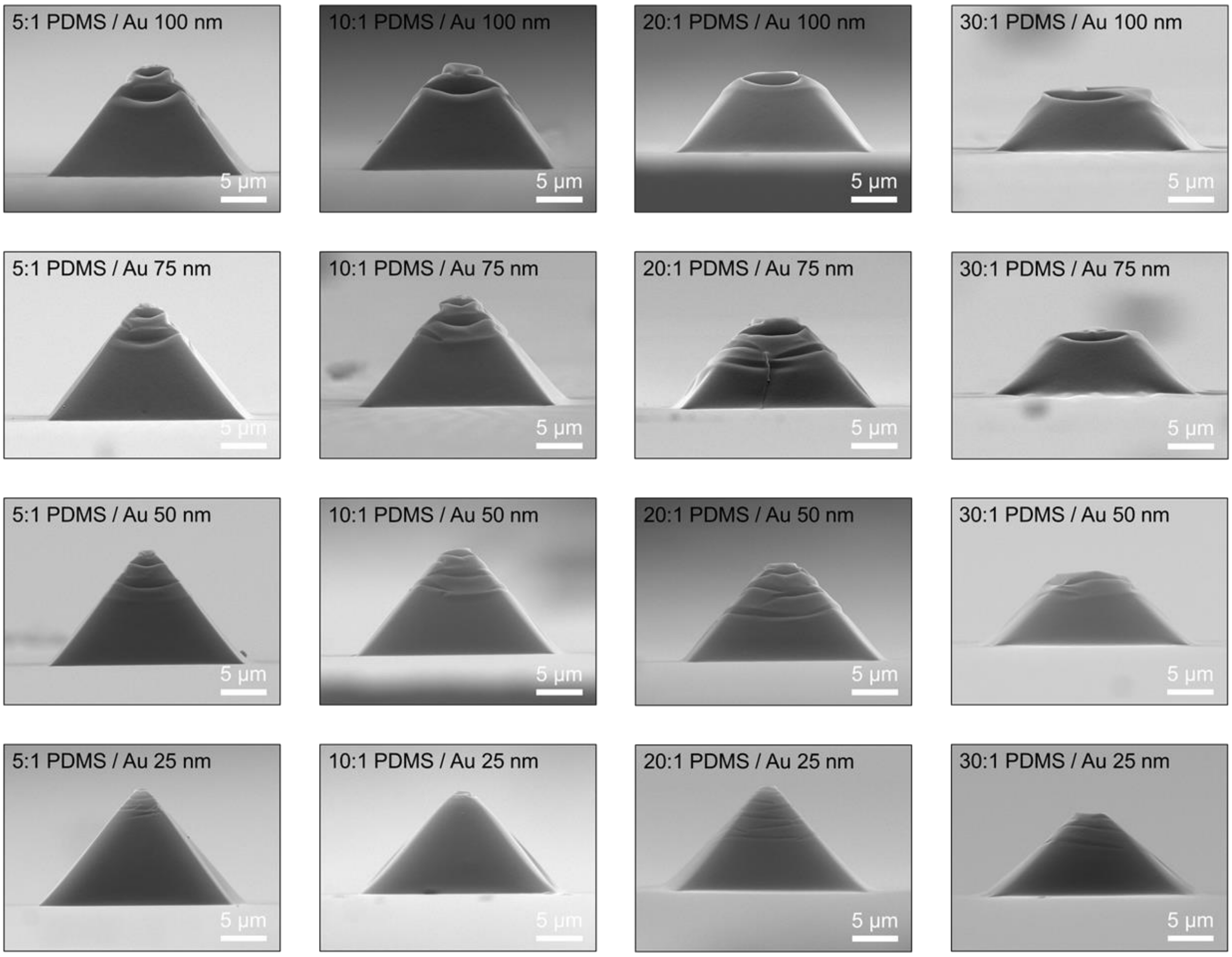

Supplementary Fig. 8 | SEM images of the metal-coated elastomer tip after the compression-relaxation test.

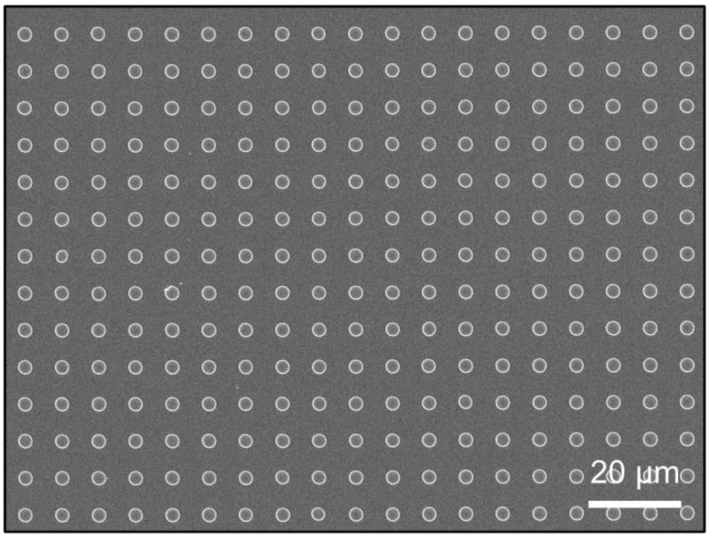

Supplementary Fig. 9 | SEM image of the 4-µm-diameter circle patterns.

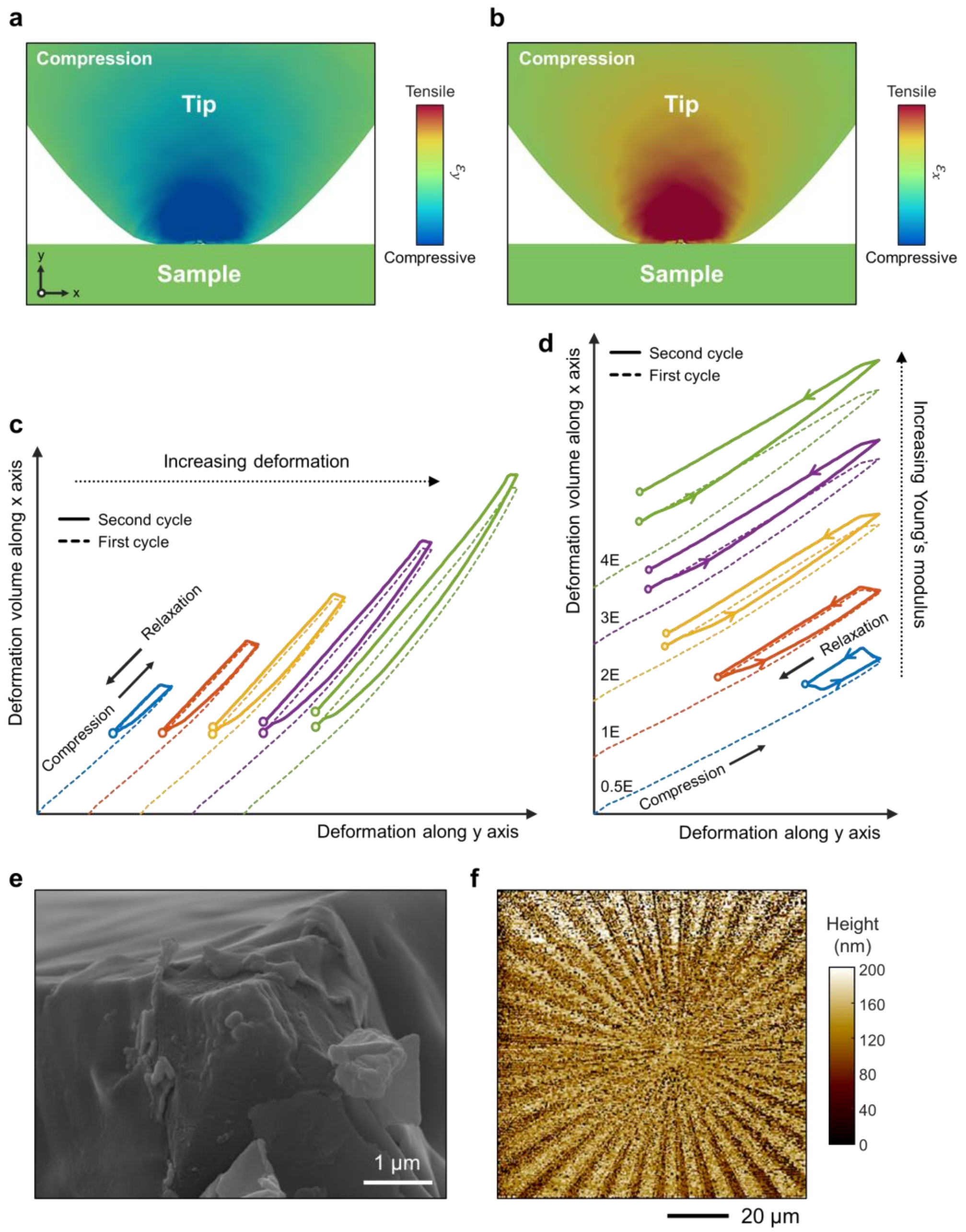

Supplementary Fig. 10 | Mechanical stability of the gold film on the elastomeric body. a-b, Simulated results of the out-of-plane (y-axis, **a**) and in-plane (x-axis, **b**) stress distributions in the compressed metal-coated elastomer composite. **c-d,** Simulated results of the deformation volume of the gold film along the x-axis according to the deformation along the y-axis with variation in the magnitude of the vertical deformation (**c**) and Young's modulus of the elastomeric body (**d**) during the two compression-relaxation cycles. The deformation volume indicates the integration of the strain of the gold film along the in-plane direction over the total

volume. The graphs in **c** and **d** indicate that the gold film lost the cyclability of the deformation volume along the in-plane direction as the deformation along the out-of-plane direction and Young's modulus of the elastomeric body increased. Losing the cyclability means that the gold film was permanently elongated along the in-plane direction each time the gold film experienced the compression-relaxation cycle, which could result in a failure in the gold film during repeated contact with the surface. **e,** SEM image of the failed gold film on the metal-coated elastomer tip (5:1 PDMS/Au 25 nm) after the BSPM measurement. **f,** Topography image of the Siemens star test chart measured by the tip under 5:1 PDMS/Au 25 nm.

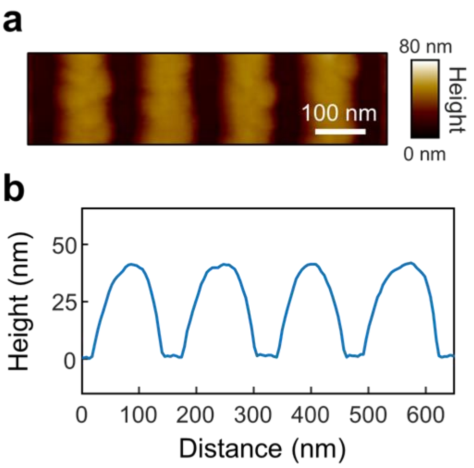

Supplementary Fig. 11 | AFM measurements of the resolution test patterns. a-b, AFM image (**a**) and line profile (**b**) of the features with a width of 83 nm and a thickness of 40 nm.

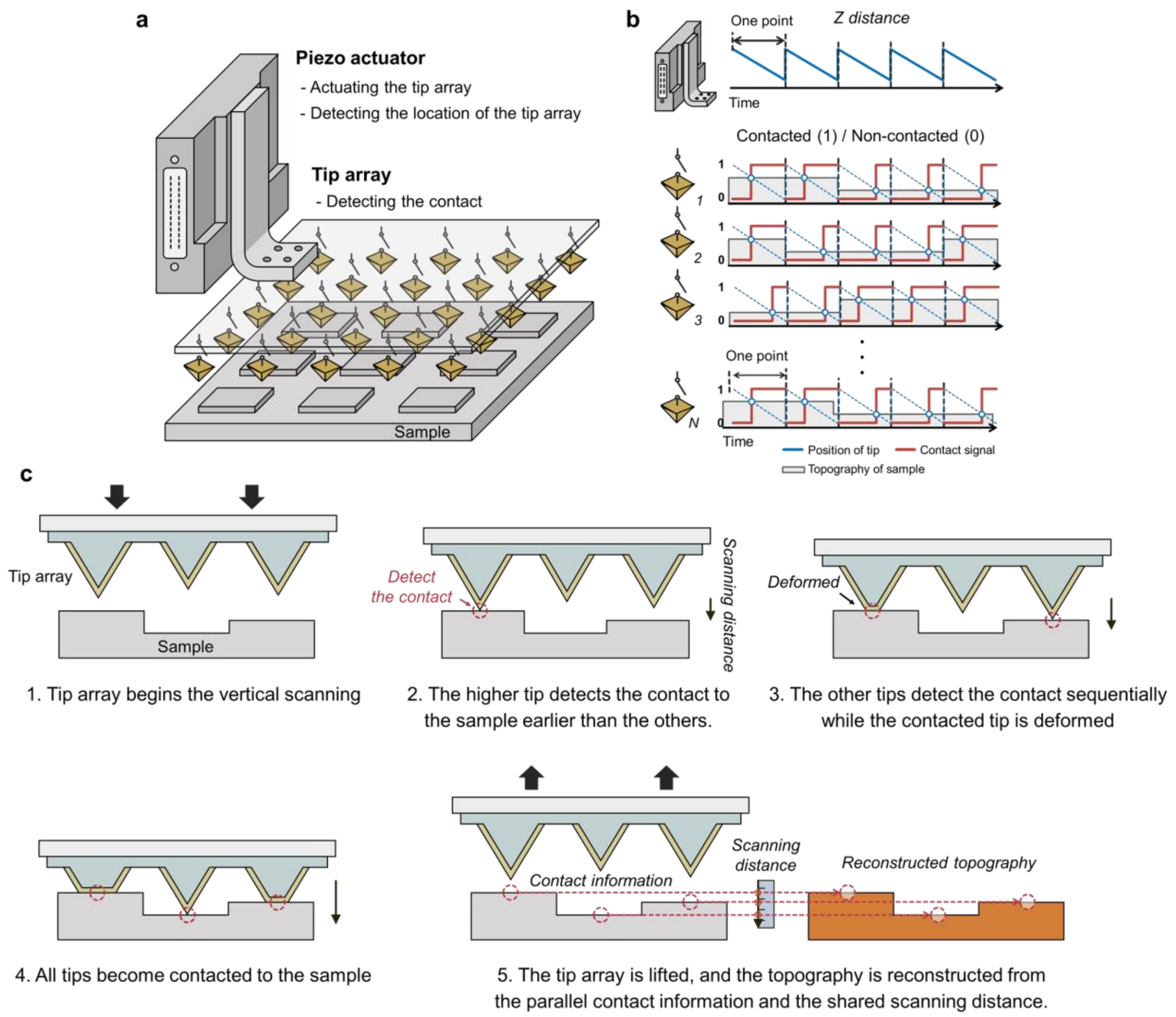

Supplementary Fig. 12 | Shared architecture of the BSPM measurement. a, Schematic illustration of the multiple-tip operation of the BSPM measurement. **b,** Graphs conceptually depicting the parallelized measurement of the BSPM system. **c,** Illustrations depicting the procedure of the BSPM measurement using a parallel tip array.

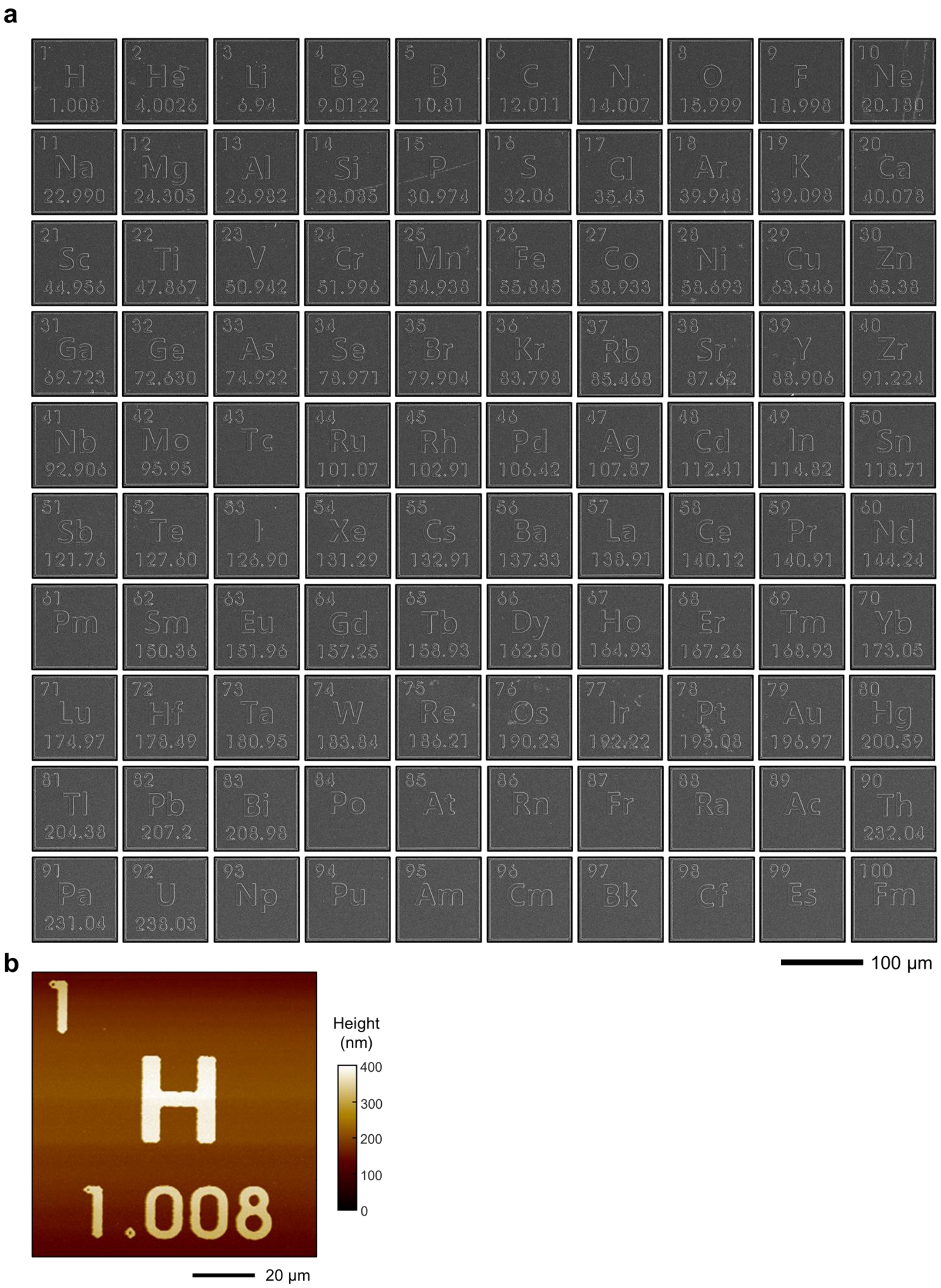

Supplementary Fig. 13 | Periodic table patterns. a, SEM images of the periodic table patterns. **b,** AFM image of the hydrogen portion of the periodic table patterns with a thickness of 175 nm.

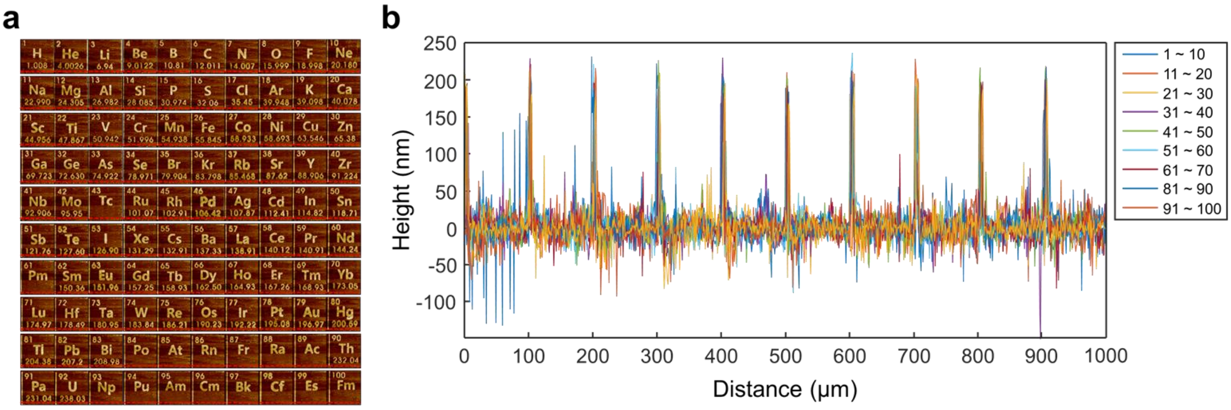

Supplementary Fig. 14 | Evaluation of the precision of parallelized measurement using the 100-tip array. a, Position of line profiles indicated as red dashed line. **b,** Line profiles of parallelized measurement using the 100-tip array to evaluate measurement precision.

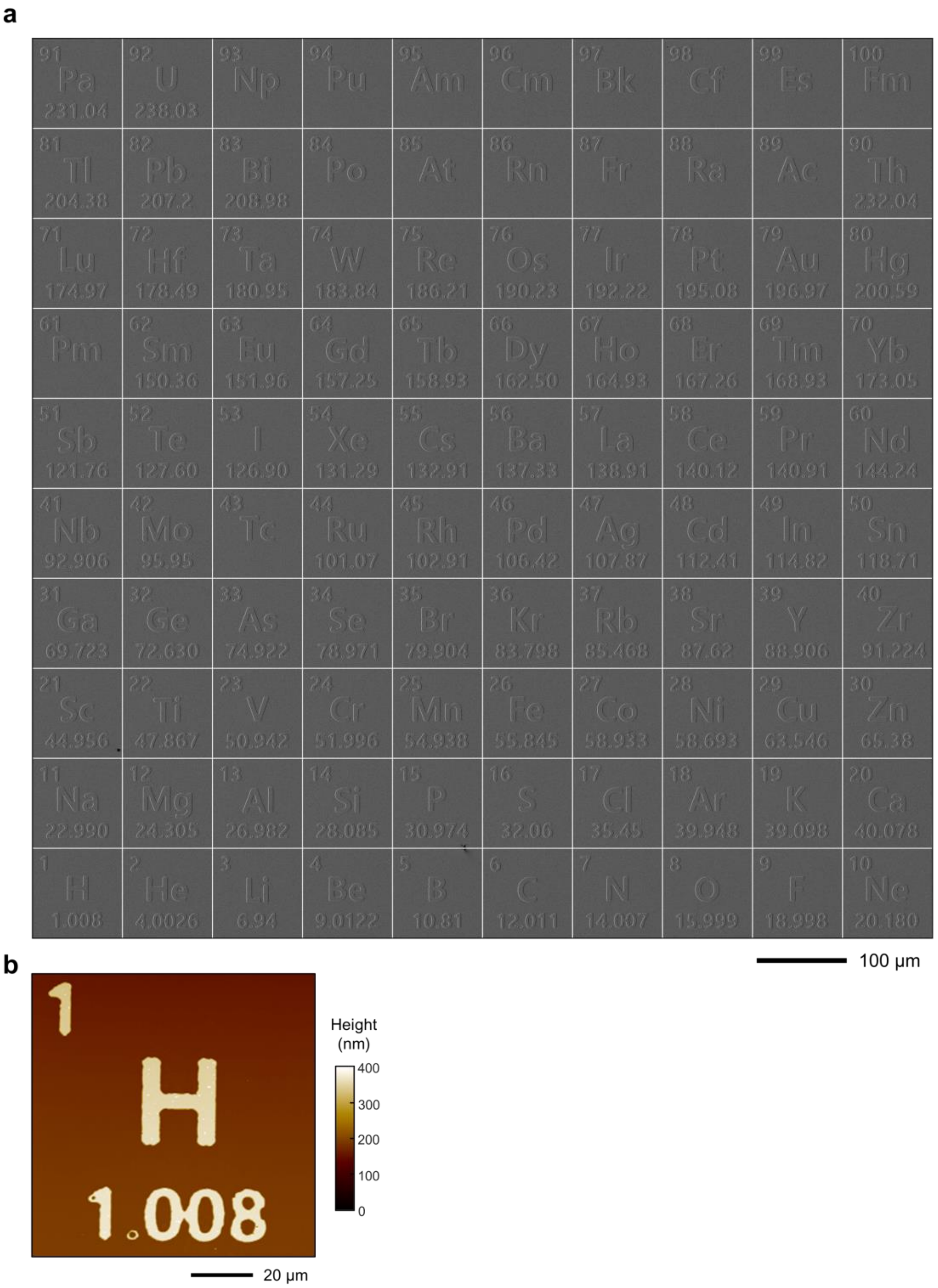

Supplementary Fig. 15 | Periodic table patterns for the parallel measurement using a 10 × 10 tip array. a, SEM images of the periodic table patterns. **b,** AFM image of the hydrogen portion of the periodic table patterns with a thickness of 177 nm.

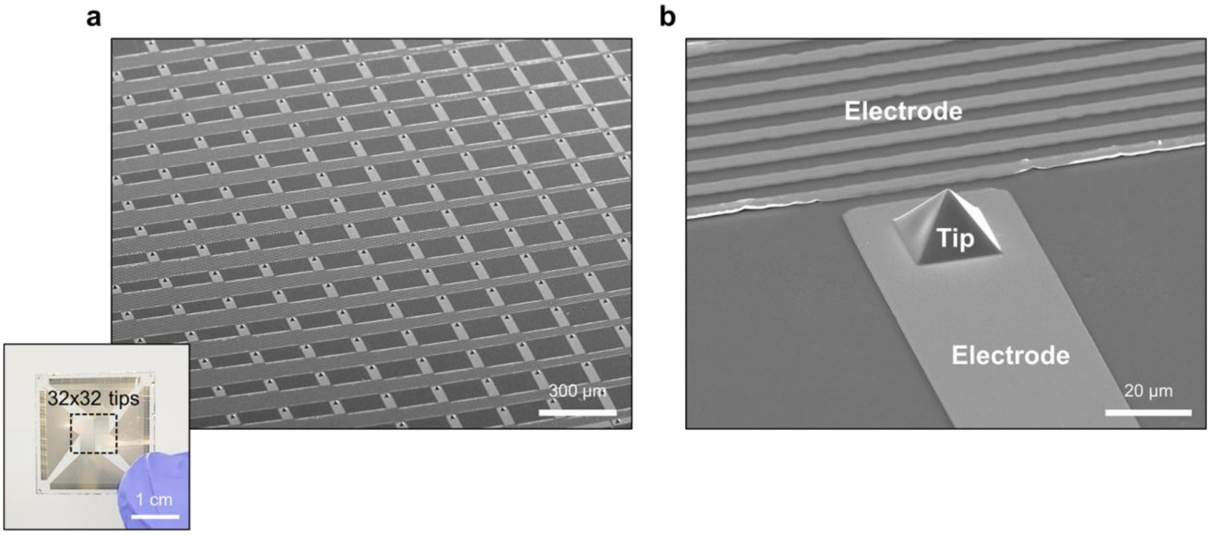

Supplementary Fig. 16 | 32 × 32 tip array. a, SEM image and photograph of the 32 × 32 tip array. **b,** SEM image of the tip and the electrodes in the 32 × 32 tip array.

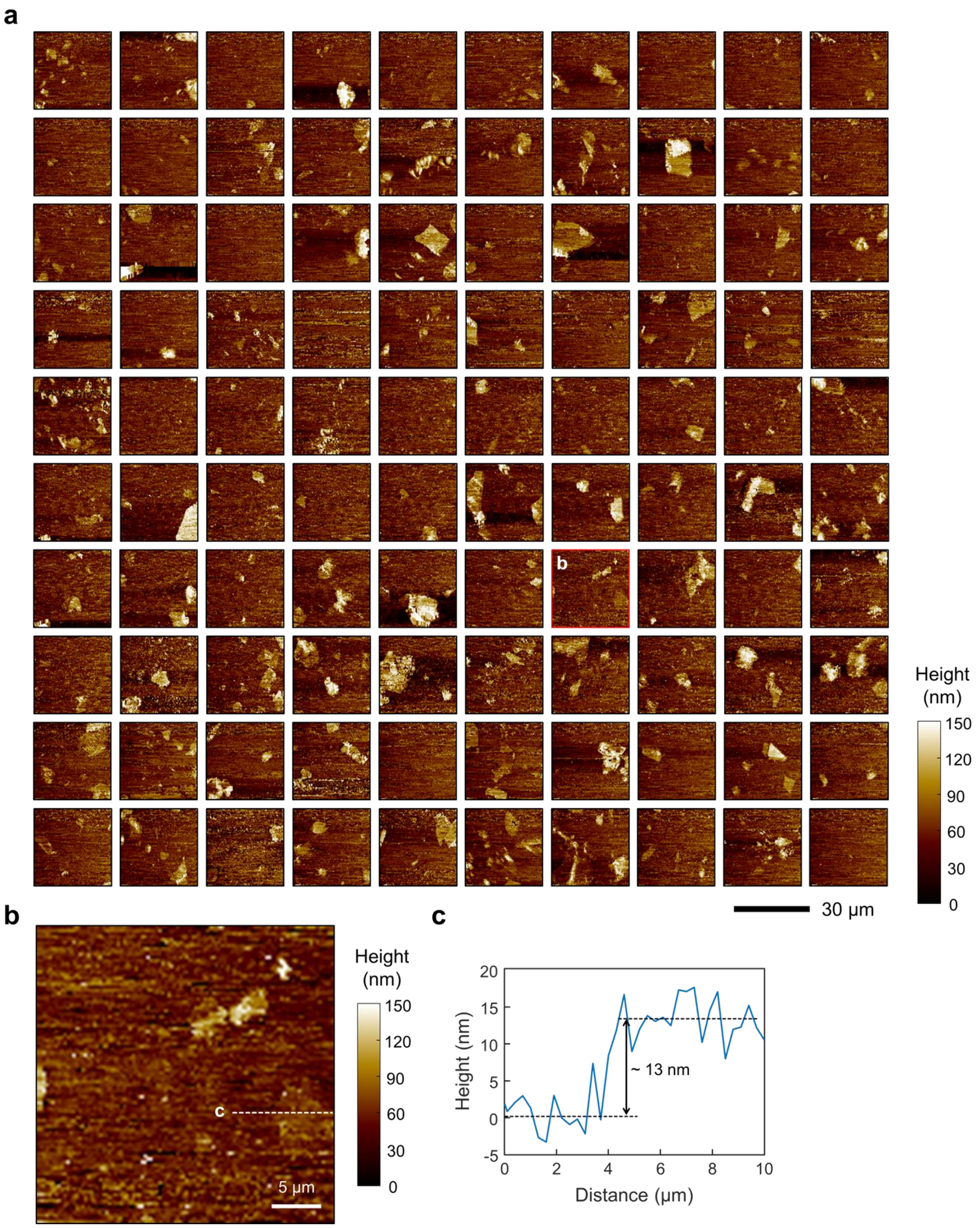

Supplementary Fig. 17 | BSPM measurement of the multilayer graphene sheets using the one-hundred-tip array. a, 100 images of the multilayer graphene sheets on the silicon substrate measured by the one-hundred-tip array. **b-c,** Topography image (**b**) and line profile (**c**) of the thinnest multilayer graphene sheet, with a thickness of 13 nm.

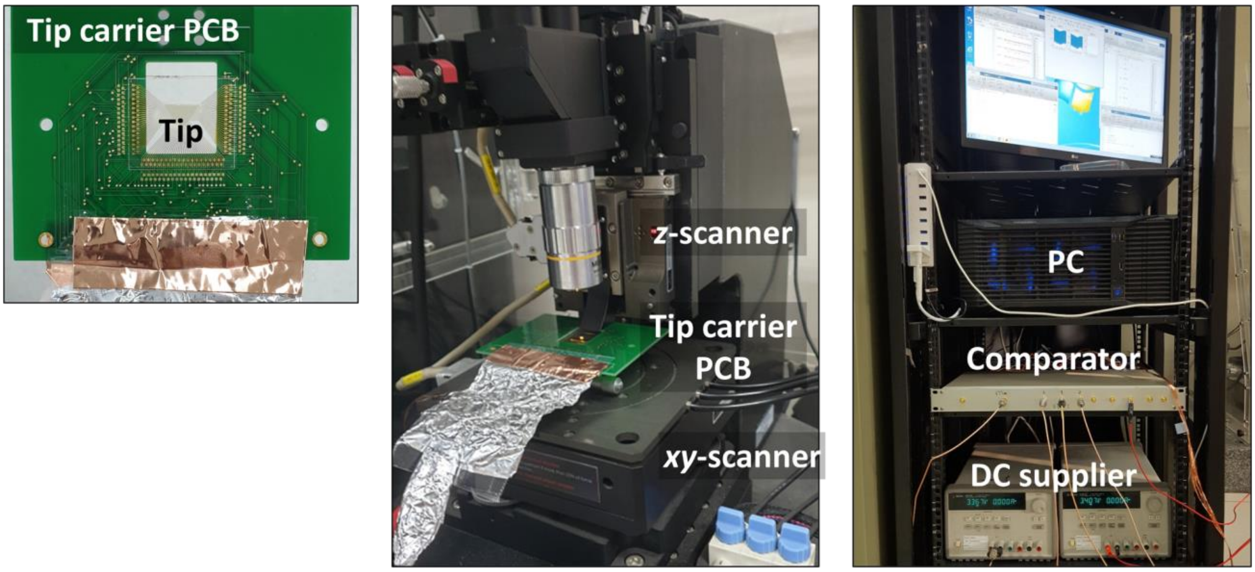

Supplementary Fig. 18 | Instrument setup for BSPM measurement.

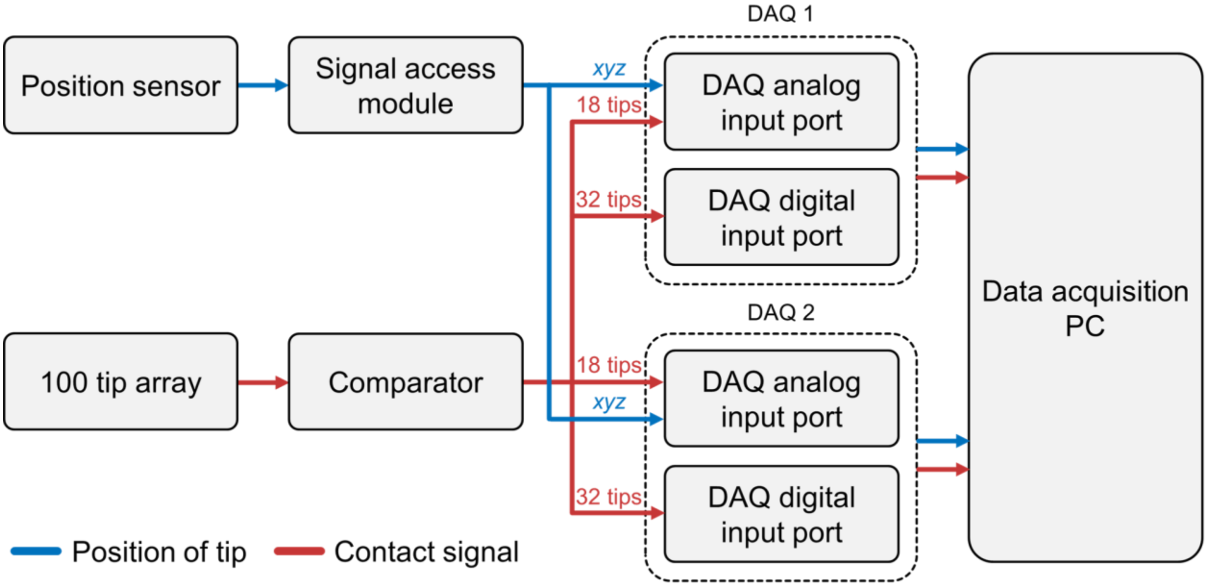

Supplementary Fig. 19 | Diagram of the data acquisition flow of 100 tip array measurement.

Supplementary Table 1 | Summary of the imaging capabilities.

	This work	
	**Single**	**Parallel (100 tips)**
**Vertical resolution**[1)]	1.8 nm	13 nm
**Vertical precision**[2-1)]	9.6 nm<br>(R_{sample}[2-2)] = 5.7 nm)	16 nm<br>(R_{sample}[2-2)] = 3.4 nm)
**Lateral resolution**[3)]	83 nm	780 nm
**Scan area**	100 × 100 µm^2	1 mm^2
**Imaging rate**[4)]	1,700 Hz<br>(1,000,000 points per 589 s)	
**Processing rate**[4)]	203,000 points per second	

[1)] Defined as the height of the lowest feature. Data from Fig. 2i and Fig. S17c.

[2-1)] Defined as the standard deviation of data on the flat regions. Data from Fig. S5 and Fig. S14b.

[2-2)] R_{sample} is the root-mean-squared roughness of sample.

[3)] Defined as the width of the smallest feature. Data from Fig. 3m and Fig. 5e.

[4)] Parallel measurement condition.

Supplementary Text 1. Contact behaviour of the metal-coated elastomer tip

Because the BSPM measurement scans the surface without force feedback, vertical scanning of the tip could lead to mechanical interaction between the tip and the sample after electrical contact detection. The magnitude of deformation during vertical scanning is an important criterion for estimating the proper mechanical properties of the sample for BSPM measurement, and thus the detailed contact behaviour should be investigated.

To analyse the contact behaviour of the metal-coated elastomeric tips, we performed a 3D finite element method (FEM) simulation. In this simulation, we modelled the geometry of the tip as a pyramid with a height of 14 μm and set the Au film thickness as 50 nm. The Young's moduli of the elastomeric body, metal film, and sample were 1 MPa, 70 GPa, and 1 GPa, respectively. The Poisson ratios of the elastomeric body, metal film, and sample were 0.49, 0.42, and 0.49, respectively. The elastic limit of the metal film was set to 0.67%, and we assumed that the sample exhibits linear elastic behaviour.

Supplementary Fig. 20 shows the simulated results of a typical contact process between the tip and sample. The contact phenomena of a metal-coated elastomeric tip include indentation and deformation steps, depending on the scanning distance. At the beginning of contact, the metal film on the tip acted as a hard tip and indented the surface within a short distance. Then, the contact deformation exceeded the elastic limit of the Au film, and the tip behaved like an elastomeric tip. At this step, the contact force was relieved by deforming the tip itself, and thus sample deformation sharply decreases (Supplementary Fig. 20b).

Next, we used FEM simulations to quantify the sample deformation with respect to the mechanical properties of the sample and Au film (Supplementary Fig. 20c). This simulation estimates the degree of sample deformation at a scanning distance of 100 nm for different Young's moduli of the sample and thicknesses of the Au film. As a result, it was found that sample deformation decreased with increasing Young's modulus. For the sample with a

Young's modulus greater than $\approx 10^3$ MPa, the sample deformation becomes less than one-tenth of the scanning distance. In addition to the stiffness of the sample, the thickness of the Au film also affects the sample deformation.

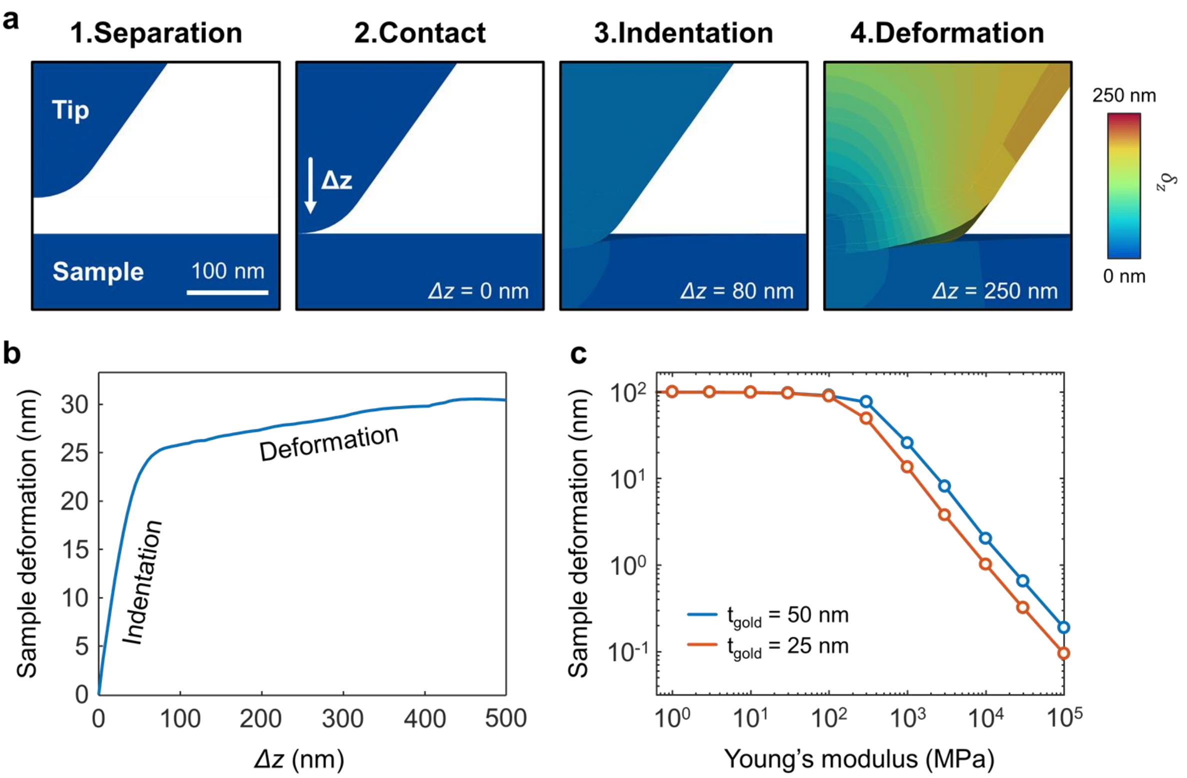

Supplementary Fig. 20 | Analysis of contact behaviour between a metal-coated elastomeric tip and sample. a, Contact processes during vertical scanning with simulated z-displacement (δ_z) distribution at each step. **b,** Simulated result of sample deformation with increasing vertical scanning (Δz). **c,** Simulated result of sample deformation at Δz = 100 nm for different Young's moduli of sample and thicknesses of Au film.

Supplementary Text 2. Mechanical crosstalk between neighbouring tips

Considering that each tip in the array is mechanically coupled with neighbouring tips through a thin PDMS layer (4 μm), it is reasonable to expect that the large deformation of the tip may distort the position of neighbouring tips. To estimate the mechanical crosstalk between the neighbouring tips during vertical scanning, we performed an FEM simulation (Supplementary Fig. 21). In this simulation, we set the height of the metal-coated elastomeric tip to 14 μm and the tip-to-tip distance to 100 μm, which is consistent with the experimental conditions. In order to solely evaluate the contact behaviour of the tip, this simulation assumes the sample to be a rigid body. We observed the z-displacement of the backing layer and the neighbouring tip when the tip was deformed with a scanning distance of 1 μm.

Supplementary Fig. 21a shows the simulated results of the z-displacement in the tip array. Most of the deformation occurs in the vicinity of the tip apex, and thus the strain propagation to the backing layer is limited. The z-displacement of the backing layer was less than 100 nm at a scanning distance of 1 μm (Supplementary Fig. 21b). As a result, the mechanical crosstalk of neighbouring tips through the backing layer is negligible, indicating that each tip in the array is well isolated to work independently (Supplementary Fig. 21c).

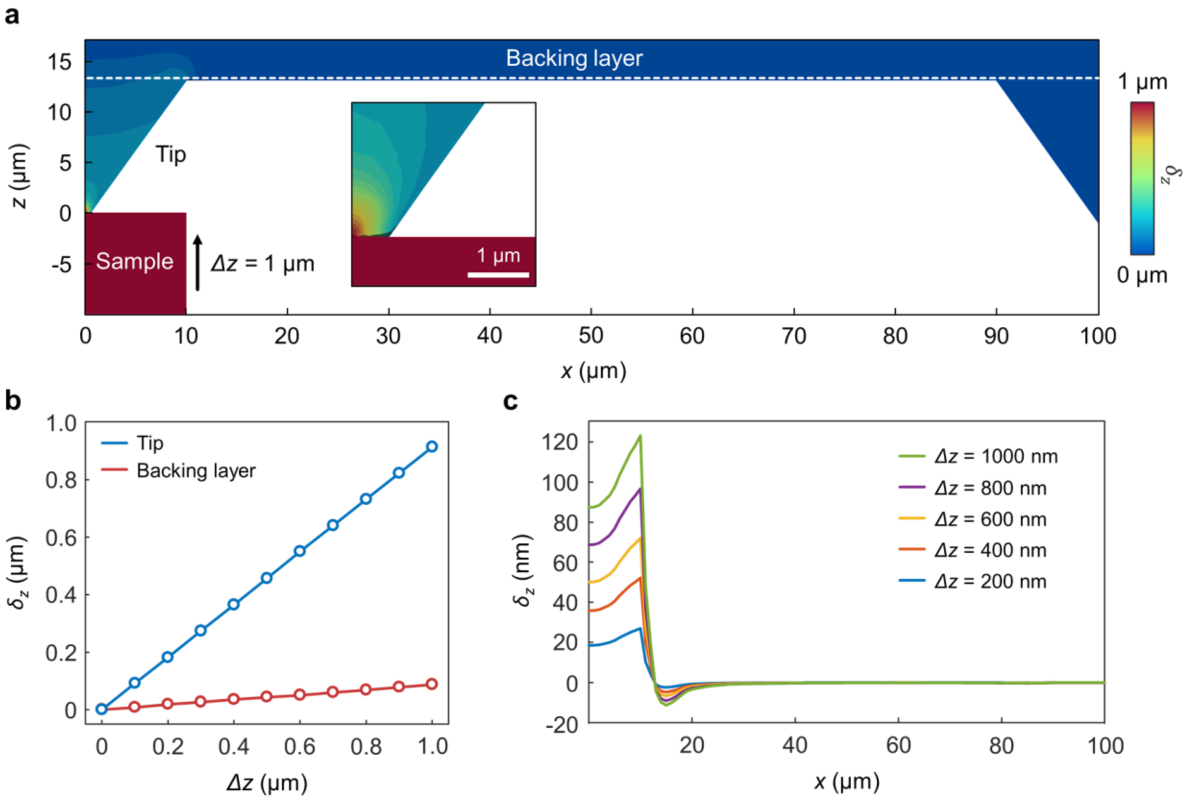

Supplementary Fig. 21 | Mechanical crosstalk between neighboring tips. a, Simulated result of z-displacement (δ_z) distribution in metal-coated elastomer tips and backing layer at a scanning distance (Δz) of 1 μm. **b,** Simulated result of z-displacement of the tip and backing layer depending on the scanning distance **c,** Simulated z-displacement profile in the backing layer (dashed line in a) for different scanning distances.
