# Supplementary material for: Binary-state scanning probe microscopy for parallel imaging
Source: Nat Commun. 2022 Mar 17;13:1438. doi: 10.1038/s41467-022-29181-z (PMC8931021; doi:10.1038/s41467-022-29181-z)
Supplement: Supplementary file 2 — Description of Additional Supplementary Files [file 41467_2022_29181_MOESM2_ESM.pdf]

Title: Supplementary Video 1.

Description: Operation principle of BSPM

Title: Supplementary Video 2.

Description: Single tip scanning

Title: Supplementary Video 3.

Description: Simulation describing contact behaviour of the metal-coated elastomer tip

Title: Supplementary Video 4.

Description: Operation principle of parallel BSPM
